# Structural Requirements for Cub Domain Containing Protein 1 (CDCP1) and Src Dependent Cell Transformation

**DOI:** 10.1371/journal.pone.0053050

**Published:** 2012-12-31

**Authors:** Gwendlyn Kollmorgen, Birgit Bossenmaier, Gerhard Niederfellner, Hans-Ulrich Häring, Reiner Lammers

**Affiliations:** 1 Pharma Research and Early Development, Roche Diagnostics GmbH, Penzberg, Germany; 2 Department of Internal Medicine IV, University of Tübingen, Tübingen, Germany; Hungarian Academy of Sciences, Hungary

## Abstract

Cub domain containing protein 1 (CDCP1) is strongly expressed in tumors derived from lung, colon, ovary, or kidney. It is a membrane protein that is phosphorylated and then bound by Src family kinases. Although expression and phosphorylation of CDCP1 have been investigated in many tumor cell lines, the CDCP1 features responsible for transformation have not been fully evaluated. This is in part due to the lack of an experimental system in which cellular transformation depends on expression of exogenous CDCP1 and Src. Here we use retrovirus mediated co-overexpression of c-Src and CDCP1 to induce focus formation of NIH3T3 cells. Employing different mutants of CDCP1 we show that for a full transformation capacity, the intact amino- and carboxy-termini of CDCP1 are essential. Mutation of any of the core intracellular tyrosine residues (Y734, Y743, or Y762) abolished transformation, and mutation of a palmitoylation motif (C689,690G) strongly reduced it. Src kinase binding to CDCP1 was not required since Src with a defective SH2 domain generated even more CDCP1 dependent foci whereas Src myristoylation was necessary. Taken together, the focus formation assay allowed us to define structural requirements of CDCP1/Src dependent transformation and to characterize the interaction of CDCP1 and Src.

## Introduction

The Src tyrosine kinase is overexpressed in many tumor types, and its kinase activity may increase as the tumor stage advances [1], [2]. This is, however, not correlated with increased cell proliferation, and even cooperation of Src with the epidermal growth factor receptor rather enhances cell invasiveness [3]. The kinase activity of c-Src is regulated by two phosphorylation events: the carboxy-terminal tyrosine residue (Y529 in mouse) is phosphorylated by the C-terminal Src kinase (CSK) and then bound by the Src-SH2 domain. The binding leads to a closed conformation and prevents ATP to access the catalytic center of the kinase domain. Dephosphorylation of Y529 by a protein tyrosine phosphatase transiently allows the conformation to open. Full activation is obtained by phosphorylation of Y418 that stabilizes the activation loop of the kinase and thus the open conformation of the catalytic cleft (reviewed in [4], [5]). While activation of c-Src is tightly regulated this is not possible for v-Src, because the protein generated by the Rous sarcoma virus lacks the carboxy-terminal sequence containing the negative regulatory tyrosine residue. Carboxy-terminal truncation of Src is also found in rare cases of human tumors [6].

Many aspects of Src activation are still unknown, for example the role of transmembrane proteins providing docking sites in the activation process. The Src family kinase member Lck binds via a di-cysteine motif to the T-cell proteins CD4 and CD8 [7]. Although the exact sequence of events is unclear, phosphorylation of the carboxy-terminal tyrosine residue (Y505) in this associated pool of Lck is tightly regulated by the tyrosine phosphatase CD45 [8]. Binding of CD4/CD8 to the major histocompatibility complex of a neighboring cell clusters CD4/CD8 proteins and brings the associated Lck molecules in close proximity so that they can phosphorylate and activate each other. In a similar activation model, the Src family kinase members Fyn and Yes bind Nephrin, a protein expressed in podocytes of the kidney glomeruli. This interaction is mediated by their SH3 domains [9]. Clustering by engagement of the Nephrin extracellular domains also leads to Fyn kinase activation [10].

Recently, a Src kinase docking protein, Cub domain containing protein 1 (CDCP1), has been described. This protein is phosphorylated by Src [11] and bound by the Src-SH2 domain [12]. CDCP1 was identified independently by several groups. Scherl-Mostageer et al. [13] found it, because it is overexpressed in lung and colon cancer, whereas Hooper et al. [14] and Yang et al. [15] showed that it was overexpressed in metastatic cells, and Bhatt et al. [16] identified it as a cell cycle dependent substrate of the Src kinase. Bhatt et al. also showed that CDCP1 interacts with several matrix and transmembrane proteins and is a substrate of the MT-SP1 protease. Overexpression of CDCP1 in tumors may be a prognostic marker for survival [17], [18]. Recent data by Gioia et al. [19] show that it is also upregulated in nilotinib resistant chronic myeloid leukemia cells. While there are numerous studies on the expression of CDCP1 in tumors or tumor derived cell lines, little is known about the CDCP1 - Src signaling complex and its regulation or how their interaction could promote cell transformation. Brown et al. [11] identified Y734 as the major phosphorylated residue in CDCP1. Another important phosphorylated tyrosine is Y762 that is bound by protein kinase C (PKC) δ [12]. PKCδ is required for migration [20] and may play a role for anoikis resistance in lung adenocarcinoma [21].

In the present manuscript, we infected NIH3T3 cells with a combination of retroviruses encoding c-Src and CDCP1 or mutated variants of them. We found that coexpression of both wild type proteins led to cell transformation, as determined by focus formation, whereas various deletions and point mutations of CDCP1 reduced transformation efficiency.

## Results

### CDCP1 and c-Src Cooperate in Fibroblast Transformation

**Figure 1:**
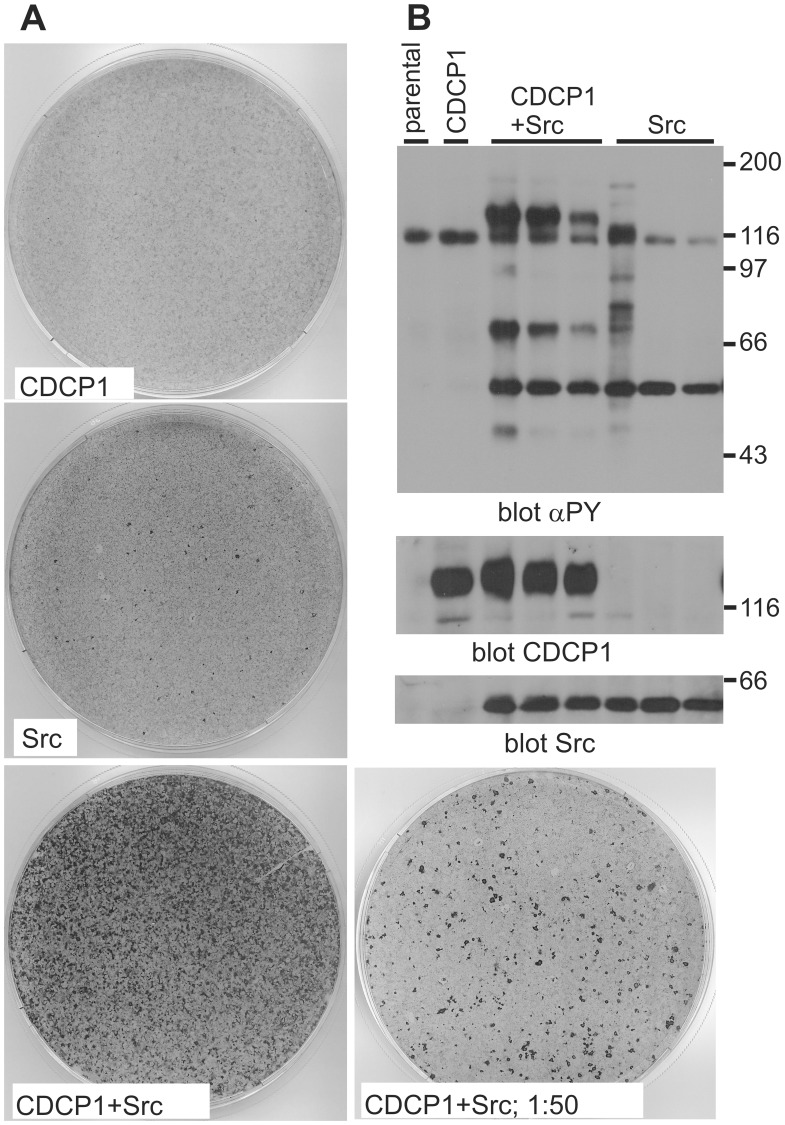
Co-overexpression of Src and CDCP1 leads to focus formation. (A) Fifty thousand NIH3T3 cells were infected with 500.000 retroviruses encoding CDCP1 or Src individually or together. After 48 h the cells were reseeded in a 10 cm-dish and grown for 3 weeks, with 3 media changes per week. In parallel, a 2% aliquot was taken from the CDCP1/Src infected cells and reseeded together with 150.000 parental NIH3T3 cells (1∶50 dilution). Finally, the cells were stained with crystal violett. (B) Parental NIH3T3 cells, cells infected with the CDCP1 encoding retrovirus or individual foci derived from infections at lower m.o.i. than in *A* that had been isolated and expanded, were lysed, aliquots with similar amounts of protein run on an SDS-PAGE, proteins transferred to nitrocellulose and the filter blotted with the indicated antibodies. αPY, anti-phosphotyrosine. Size markers (kDa) are indicated.

To understand the signaling processes that are involved in CDCP1-mediated cellular transformation we employed a focus formation assay using a NIH3T3 cell line with a very low degree of spontaneous loss of contact inhibition. These fibroblasts have previously successfully been used to analyze oncogenic transformation, e.g. by the CSF-1R [22]. In this assay, upon infection of the cells with retrovirus encoding wild type CDCP1 we did not observe the formation of foci that appear when cells are transformed and grow on top of each other because of loss of contact inhibition. Since CDCP1 is a substrate of the Src kinase, we infected in a parallel experiment cells with two different viruses encoding the c-Src or wild type CDCP1 protein. Upon coinfection many foci were formed, whereas infection with the Src encoding retrovirus alone caused only a few foci to develop (Fig. 1A). To confirm the expression of CDCP1 and Src in the foci, three individual foci generated in infections using lower virus numbers were picked, expanded and the cell lysates analyzed by SDS-PAGE and Western blotting (Fig. 1B). As a control, parental cells and a pool of cells infected with CDCP1 wild type virus were analyzed as well. A blot with an antibody against phosphotyrosine is shown in the upper panel, the expression of CDCP1 in the middle panel and that of Src in the bottom panel. In parental cells and those expressing only CDCP1, tyrosine phosphorylation at the exposure level shown was confined to one protein of 110 kDa whereas foci derived cells showed in addition phosphorylation of Src (60 kD) and CDCP1 (130 kD) proteins. The 70 kD protein in CDCP1 and Src infected cells probably represents CDCP1 after cleavage of the extracellular domain. Of note, overexpressed CDCP1 was only tyrosine phosphorylated upon Src overexpression. This demonstrates that a coordinated co-overexpression of CDCP1 and Src is required for loss of contact inhibition and cell transformation. With this assay it was tested which domains of the CDCP1 protein are required for transformation and which role specific amino acids play.

### Tyrosine Phosphorylation of CDCP1 and Cellular Transformation

**Figure 2:**
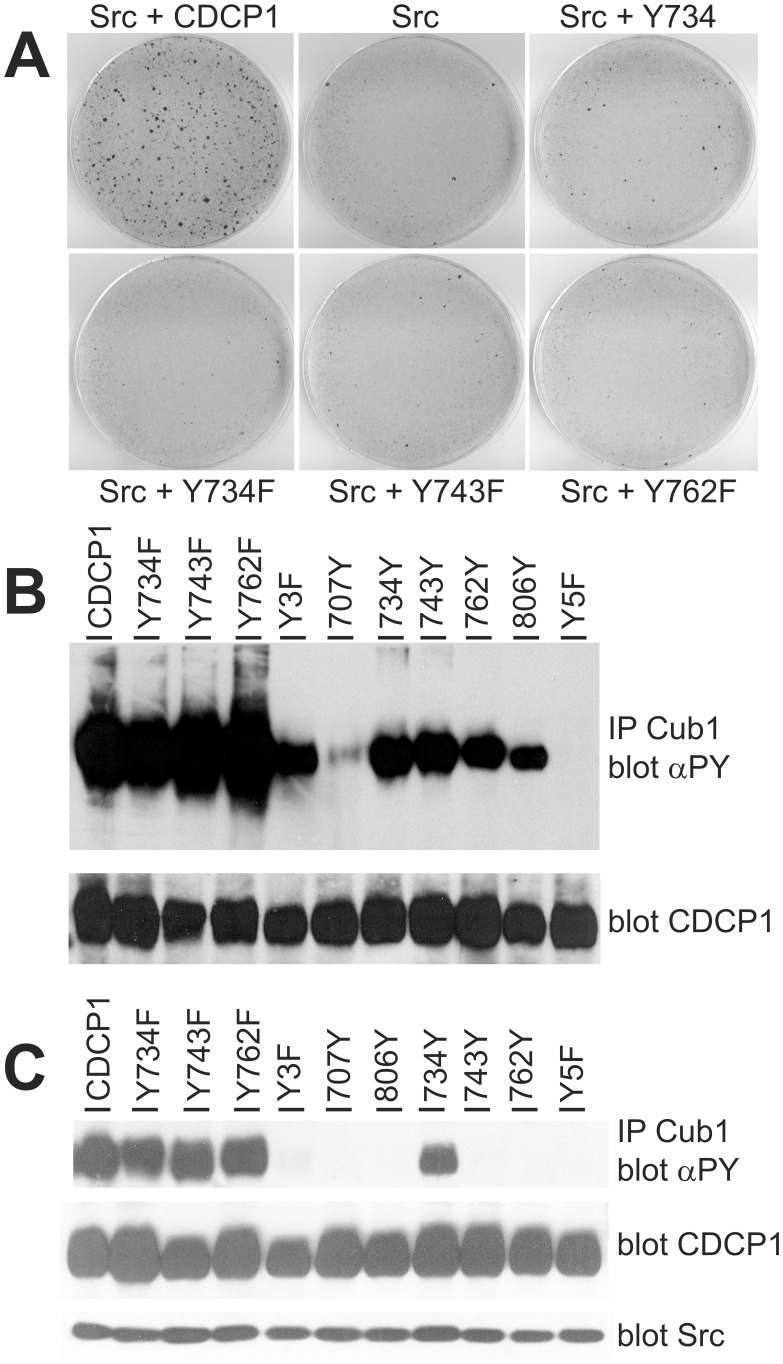
Src and CDCP1 dependent focus formation require tyrosine phosphorylation of CDCP1. (A) NIH3T3 cells were infected as above with Src alone or with Src and the indicated CDCP1 forms. After three weeks, the cells were stained with crystal violett. Y734 - all codons for tyrosine residues but Y734 were mutated to encode Phe; Y734/743/762F - the codons for these tyrosines were mutated to Phe. (B) CDCP1 and the indicated tyrosine mutants were transiently overexpressed in 293 cells. After treatment with peroxovanadate the cells were lysed and CDCP1 immunoprecipitated with the Cub1 antibody. Immunoprecipitates were analyzed by SDS-PAGE and Western blotting with a phosphotyrosine-antibody (αPY; upper panel). In parallel, an aliquot from the cell lysate was analyzed for CDCP1 expression with an antibody against CDCP1 (lower panel). (C) Similar to (B), 293 cells were transfected with plasmids encoding CDCP1 and its mutants, and in addition c-Src. Cells were analyzed as above and the Cub1 immunoprecipitates detected with a phosphotyrosine specific antibody, whereas in an aliquot of the cell lysate CDCP1 and c-Src expression were tested (lower panel). αPY, anti-phosphotyrosine.

Tyrosine phosphorylation of cell surface proteins can be essential for the formation of protein signaling complexes. Y734 in CDCP1 is a target of the Src kinase [11] and, when phosphorylated, also binds to the Src-SH2 domain [12]. Another phosphorylated tyrosine, Y762, is bound by PKCδ. Phosphorylation of Y743 has been reported to occur to a lesser extent [12]. We have tested the relevance of these phosphorylation sites for transformation by mutating the corresponding nucleotides encoding tyrosine residues in the coding sequence of CDCP1 to encode phenylalanine. Retroviral expression constructs were generated, and corresponding viruses used in focus formation assays (Fig. 2A). As above, the combined infection with Src and CDCP1 wild type retroviruses enhanced focus formation strongly. The bigger size and reduced number of foci compared to Figure 1 is the result of a reduced virus number used for infection. Co-infecting with Src and the virus encoding the Y734F mutant, the number of foci was as low as in the Src virus only infection. This was similar for viruses encoding the mutations Y743F and Y762F suggesting that all three phosphorylation sites are required for the transforming potential of CDCP1. On the other hand, a CDCP1 encoding virus where all of the intracellular tyrosines except for the residue at position 734 were mutated to phenylalanine, reproducibly yielded slightly more foci than infection with the Src virus alone or coexpression of Src and a Y/F mutant. Thus, the presence of Y734 as the only phosphorylation site retained some of the transforming capacity of CDCP1. As a control for expression of the different constructs used in this study we infected NIH3T3 cells with similar numbers of virus, selected the pools of cells for G418 resistance and analysed for expression of CDCP1 mutants (Fig. S1).

Since CDCP1 phosphorylation could play a role in its ability to overcome contact inhibition, we next analyzed the phosphorylation status of all five tyrosine residues in the CDCP1 intracellular domain. To this end, we overexpressed wild type and mutant CDCP1 containing single or different combinations of intracellular tyrosines in 293 cells, treated the cells with peroxovanadate to achieve maximal phosphorylation and immunoprecipitated CDCP1 from cell lysates (Fig. 2B). Western blot analysis with a phosphotyrosine-specific antibody showed that mutation of single tyrosine residues (Y734F, Y743F, Y762F) had little impact on total phosphotyrosine content of the protein. Using CDCP1 mutants with only one remaining intracellular tyrosine residue showed that the three central tyrosine residues (Y734, Y743 and Y762) were phosphorylated almost to the same extent, whereas Y806 was less and Y707 hardly at all phosphorylated under these experimental conditions. As a control, the mutants Y5F without any intracellular tyrosine residue or Y3F without tyrosines 734/743/762 were included and gave no or a weak signal, respectively. Since the lack of phosphorylation of Y707 conflicts with several mass spectrometric analyses that found Y707 containing phosphopeptides (http://www.phosphosite.org), we reassessed this mutant with another phosphotyrosine antibody (PY99) and readily detected Y707 phosphorylation (not shown). Thus, all intracellular tyrosines of CDCP1 can be phosphorylated, and at least Y734, Y743 and Y762 are required to achieve cellular transformation.

The Src kinase is phosphorylating Y734 in CDCP1 [12]. To determine whether Src can also phosphorylate other tyrosine residues in the CDCP1 intracellular domain, we co-overexpressed Src with the same set of CDCP1 and its mutants. Under these experimental conditions, Src was constitutively active. Interestingly, the Y734F mutant was still strongly phosphorylated at other tyrosine residues, presumably by Src (Fig. 2C). However, in the context of add-back mutants with a single intracellular tyrosine only the mutant containing Y734 was phosphorylated by Src. This proves a strong preference of the Src kinase for Y734 and, together with data generated by the peroxovanadate stimulation, points to additional kinases that are activated by Src and phosphorylate CDCP1.

In addition to the binding of Src, a phosphotyrosine mediated interaction of CDCP1 with other proteins may be important for cellular transformation. The finding that the Y762F mutant did not yield foci could support a role of PKCδ binding to CDCP1 for transformation in NIH3T3 cells. However, in experiments, in which the cells were coinfected with viruses encoding Src, CDCP1, and PKCδ wild type, kinase inactive or constitutively activated (A147E) variants, no significant changes in focus formation were observed (Fig. S2A).

### Contribution of Protein Domains to Transformation

**Figure 3:**
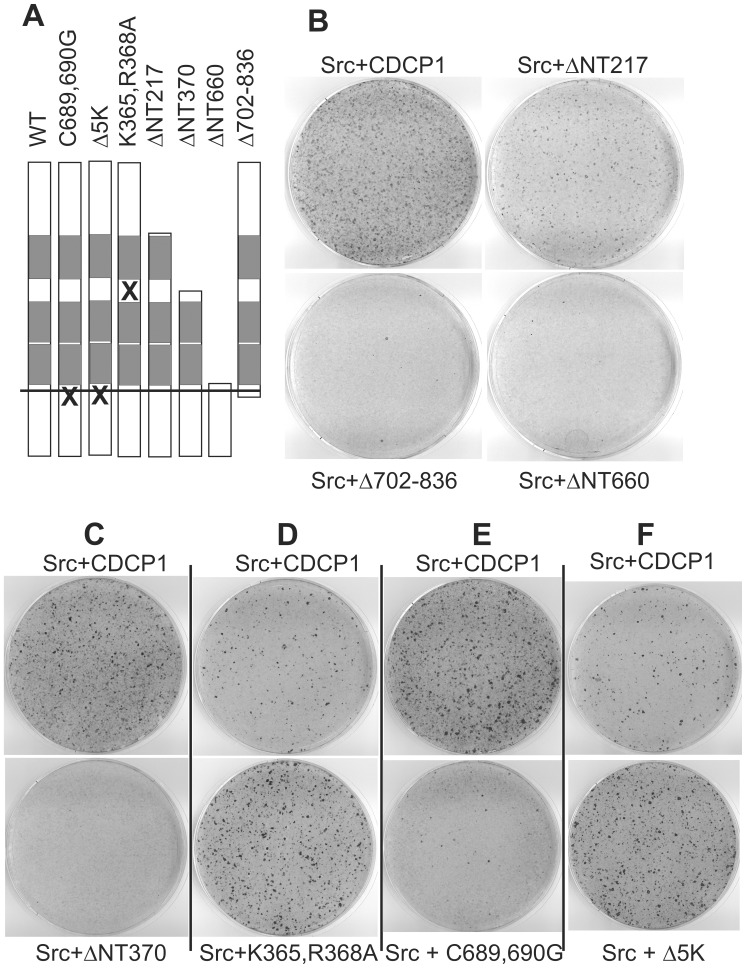
Role of CDCP1-domains for cellular transformation. (A) Schematic presentation of the mutants used. (B–F) NIH3T3 cells were infected with Src and CDCP1 or the indicated deletion mutants, grown and stained as above. In separate experiments, the mutants ΔNT370, K365A,R368A, C689,690G and Δ5K were compared in their transformation efficiency to wild type CDCP1. Please note, that the number of CDCP1 virus in (D) was reduced in comparison to the other experiments.

We next tested the contribution of the CDCP1 extracellular domain to cell transformation. The presence of Cub domains indicates that CDCP1 may be able to bind a ligand, as has been suggested early on for Cub domains [23], and this could contribute to transformation. Thus, we generated constructs with deletions of either the amino-terminus (ΔNT217, retaining the signal peptide and starting at amino acid 217 preceeding a Cub domain), or the complete extracellular domain (ΔNT660) or most of the intracellular domain (Δ702–836; Fig. 3A). As shown in Figure 3B, the lack of the complete extra- or intracellular domain abolished formation of foci. On the other hand, deletion of only the portion of the extracellular domain preceeding the Cub domain led to a reduced but distinct focus formation. This indicates that the amino-terminal 216 amino acids are involved in the transformation process but not essential. Further reducing the size of the extracellular domain by another 153 amino acids results in a protein (ΔNT370) that lacks the amino-terminal Cub domain and resembles CDCP1 after proteolytic processing by Matriptase (MT-SP1; Fig. 3C) [16]. This ∼70 kD form is found in many tumor derived cell lines [24]. However, the ΔNT370 variant was no longer transforming in the focus formation assay, indicating a requirement for the amino-terminal Cub domain. We then also mutated the MT-SP1 cleavage sequence so that the processed form can no longer be generated (Fig. 3D). Compared to the infections in Fig. 3B and C, the number of retrovirus encoding wild type or mutated CDCP1 was reduced in 3D. Here, the processing mutant had an increased potential to transform. Thus, these variants point towards a negative role of proteolytical processing for cellular transformation.

The transmembrane domain of CDCP1 ends in two Cys-residues, as calculated by the TMHMM-Server (http://www.cbs.dtu.dk/services/TMHMM-2.0/), that are likely to be palmitoylated and may predict the association of CDCP1 with lipid rafts. In addition, there is evidence that palmitoylation of Cys-Cys sequences is required for protein stability [25]. This motif in CDCP1 is followed by a Val and then by six Lys-residues that are reminiscent of K-Ras4B, whose binding to the membrane is modified by this basic sequence. To test whether these motifs affect transformation, we generated mutations changing the codons for Cys to Gly or reducing the number of Lys to one. Employment of these mutants in the focus formation assay yielded only a few colonies for the Cys-mutant (Fig. 3E) but slightly improved focus formation for the lysine deletion mutant (Fig. 3F).

Finally, to analyze the contribution of CDCP1 carboxy-terminal sequences of the intracellular domain that contain proline rich motifs (aa 773–799), we generated a construct lacking 74 amino acids from the carboxy-terminus. It still includes the Y762 but does not bind PKCδ any more. This mutant was still able to generate foci but two to three times less efficient than wild type (Fig. S2B). In summary, sequences of CDCP1 essential for cellular transformation include the most amino-terminal Cub domain and the phosphorylated tyrosine residues. However, the very amino- and carboxyterminal ends are required for realizing the full transformation capacity.

Many tumor cell lines produce a CDCP1 splice variant that is secreted and encodes the first 341 amino acids, with the carboxy-terminal two amino acids being variant specific [26]. To see whether this protein has tumor promoting effects in the NIH3T3 cell system, we generated a specific retrovirus and employed it either alone, together with Src or Src plus CDCP1 in focus formation assays (data not shown). No effect on the efficiency of focus formation was observed. Complementary, we added recombinant extracellular domain (1 µg/ml every other day) to the focus formation assay but also observed no changes.

### CDCP1 is Present as a Dimer at the Cell Surface

**Figure 4:**
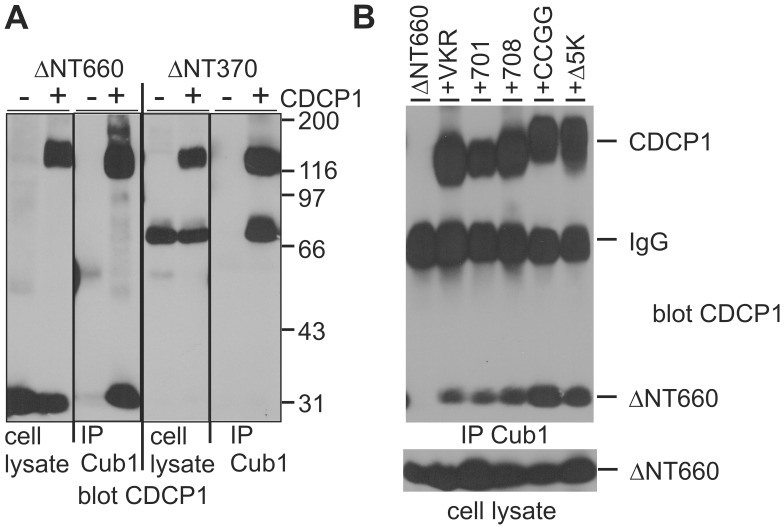
Analysis of CDCP1 dimer formation. HEK293 cells transiently overexpressing the indicated CDCP1 variants were lysed, aliquots of the lysates used for expression analysis and the rest immunoprecipitated with the antibody Cub1. Proteins were size separated by SDS-PAGE, transferred to nitrocellulose and blotted with antibodies against the intracellular (A) or the intra- and the extracellular domain (B). In *B*, size markers are indicated. VKR - CDCP1 (1–690, then Val-Lys-Arg-Stop); 701 - CDCP1 (1–701); 708 - CDCP1 (1–708); CCGG - CDCP1 (C689,690G); Δ5K - CDCP1 (ΔK693–697).

The extracellular domain specific Cub1 antibody cannot bind amino-terminally truncated CDCP1, however, the immunoprecipitate from CDCP1 expressing cell lines also contained the processed 70 kDa form (not shown). We therefore tested the possibility that CDCP1 may occur as a dimer and overexpressed the ΔNT370 mutant alone or together with wild type CDCP1 (Fig. 4A). In addition, the ΔNT660, lacking the entire extracellular domain, was included to narrow down the region of the protein responsible for interaction. Immunoprecipitation with the Cub1 antibody and analysis by SDS-PAGE and Western blotting detected the truncated forms only upon coexpression with full length CDCP1, indicating that Cub1 binds to the aminoterminus of the protein, that CDCP1 occurs indeed as a dimer, and that dimerization is mediated by the transmembrane or intracellular domain of the protein.

To identify the domain responsible for dimerization, we co-overexpressed ΔNT660 with deletion mutants of the intracellular domain or full length proteins with mutations at the membrane proximal cysteine residues or deletions of the lysine-rich stretch right after the membrane domain and immunoprecipitated the proteins with the Cub1 antibody (Fig. 4B). The ΔNT660 protein was, as expected, not immunoprecipitated by Cub1, when expressed alone, but co-immunoprecipitated with all other CDCP1 variants tested. This indicates that likely the transmembrane domain itself is responsible for dimerization, since our results exclude a role for protein sequences derived from the intra- or extracellular domain or for cysteine mediated disulfide bridges.

### Role of Src for CDCP1 Dependent Cellular Transformation

**Figure 5:**
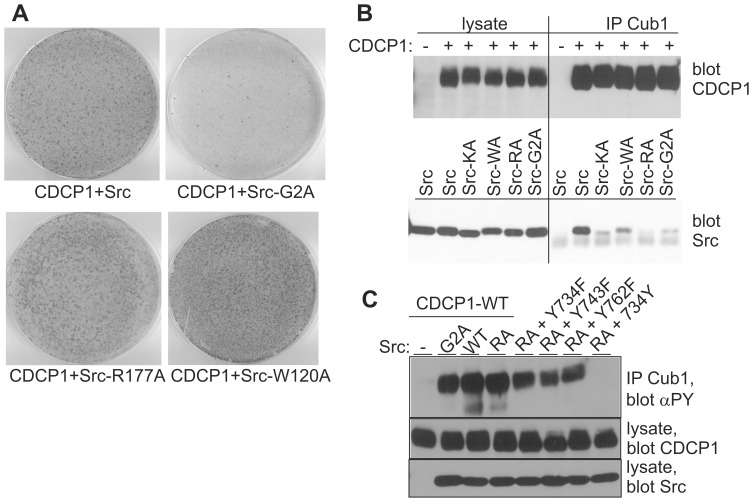
Effect of Src-mutations on CDCP1 dependent transformation. (A) NIH3T3 cells were infected with CDCP1 and the indicated Src mutants, grown and stained as described in the legend of Figure 1. (B,C) HEK293 cells overexpressing the indicated proteins were lysed, an aliquot of the lysate was used for expression analysis and the rest immunoprecipitated with the antibody Cub1. Proteins were size separated by SDS-PAGE, transferred to nitrocellulose and blotted with antibodies against CDCP1, Src or phosphotyrosine (αPY). Src-KA - kinase inactive Src; Src-WA - defective SH3 domain; Src-RA - defective SH2 domain; Src-G2A - no myristoylation.

To evaluate the features of the Src kinase for the CDCP1 dependent transformation, we generated Src mutants that could no longer be myristoylated (G2A; prevents membrane anchoring) or did not have functional SH3 (W120A) or SH2 (R177A) domains. In a focus formation assay with wild type CDCP1, the G2A mutant had reduced, while the R177A and W120A mutant had enhanced transforming capability (Fig. 5A). Co-infection of inactive Src-KA and CDCP1 did not yield foci (Fig. S3A). A slightly increased number of foci was also observed after infection with the R177A and W120A Src mutants alone (Fig. S3B) reflecting the higher basal kinase activity of these mutants. Since the Src-SH2 domain binds to phosphorylated Y734 of CDCP1 we tested whether the Src mutants still associate with CDCP1 in a transient expression system. Cell lysates and immunoprecipitates were analyzed by SDS-PAGE and Western blotting with anti-CDCP1 and anti-Src antibodies. As shown in Fig. 5B, the amount of overexpressed Src and CDCP1 proteins in the lysates were similar, however, the amount of coprecipitated Src varied depending on the Src variant. Association of CDCP1 with wild type Src was strongest, whereas association of the SH2 mutant was hardly detectable. The weak coprecipitation of the kinase inactive Src may be explained as secondary to lack of Y734 phosphorylation. However, the weak association of the G2A mutant with CDCP1 indicated an important role for Src/CDCP1 colocalization at the plasma membrane. We conclude that colocalization of CDCP1 and Src at the cell membrane is more important for cellular transformation than Src binding to CDCP1 via its SH2 domain.

We next tested, how CDCP1 phosphorylation was affected by Src mutations (Fig. 5C). Surprisingly, there was no major difference in CDCP1 phosphorylation between coexpression with Src wild type, G2A or R177A mutant. Different from the results in Figure 2, however, the CDCP1 mutant with Y734 as the only tyrosine in the intracellular domain was not phosphorylated by Src-R177A. This result, similar to the Fyn/Nephrin interaction, confirms the importance of CDCP1 clustering and strongly suggests that Y734 is required for docking of Src which then can phosphorylate the Y734 residue in the other CDCP1 protein in the cluster. The fact that in the Y734F mutant other tyrosine sites were strongly phosphorylated upon co-expression with Src-R177A, again points to a possible involvement of additional tyrosine kinases that may be activated by Src.

## Discussion

**Figure 6:**
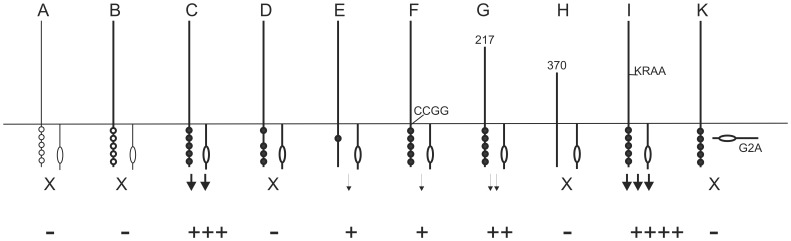
Schematic representation of major CDCP1 features important for focus formation. Only upon overexpression of Src and CDCP1, cells become transformed, and all changes in CDCP1 except for the processing mutant reduce transformation efficiency. (A–K) Left line, CDCP1 with five intracellular tyrosine residues (circles below horizontal line that represents the plasma membrane), right line with circle, Src kinase; fat print, overexpression; filled circles, tyrosine residues phosphorylated upon transient overexpression. X - no focus formation; arrows indicate focus formation. (D) mutant Y734F, (E) addback mutant Y734, (F) mutant C689, 690G, (G) mutant ΔNT217, (H) mutant ΔNT370, (I) mutant K365A, R368A, (K) myristoylation defective Src G2A mutant.

In the current manuscript, we have developed a tissue culture based model for CDCP1 mediated cell transformation that allowed us to analyze the role of several structural features of CDCP1 for Src dependent cell transformation. We characterized contributions of the extra- and intracellular domains, the proteolytic processing site, juxtamembrane sequences and individual tyrosine residues. Except for the processing mutant, all changes in CDCP1 sequence reduced transformation. CDCP1 mediated transformation itself depended crucially on co-overexpression of the Src kinase that did not need to bind directly to CDCP1 but required their association with the cell membrane. A scheme summarizing the major findings is shown in Figure 6.

Activation of Src kinases is regulated in many ways [4], [5]. In addition to its interaction with numerous receptor tyrosine kinases [27], Src also binds to several non-catalytic receptors including CDCP1 [11]. The CDCP1 initiated mechanism of kinase activation is not yet fully understood but may involve dimerization of CDCP1 which leads to clustering of the Src kinase and subsequently enhanced CDCP1 phosphorylation and Src activation, similar to CD4 clustering and Lck activation [28], [29]. We have investigated CDCP1 tyrosine phosphorylation by co-overexpression of Src or by treatment with the phosphatase inhibitor peroxovanadate. Single mutations of each of the three central intracellular tyrosine residues including Y734 did not overtly reduce phosphotyrosine content of the protein. By contrast, co-overexpression of Src with CDCP1 mutants that retained only a single intracellular tyrosine residue led to phosphorylation only in case of Y734 whereas peroxovanadate treatment induced phosphorylation of all these single tyrosines by yet unknown kinases. Nevertheless, the transforming capacity of tyrosine mutants was mostly abolished. The residual focus forming capacity of the single Y734 mutant supports its prominent role that has been described earlier [11], [30] but is not sufficient for efficient transformation and implicates additional pathways for CDCP1 signaling. Recently, Spassov et al. [31] showed that phosphorylation of the major tyrosine sites of CDCP1 inhibited cell adhesion and focal adhesion signaling by preventing integrin clustering. Also in their system, overexpression of a YΔF mutant lacking three intracellular Tyr residues was no longer able to inhibit cell adhesion.

For both, extra- and intracellular domains, a partial truncation of the protein reduced transformation efficiency. Whereas the loss of signaling capacity for the truncated intracellular domain is plausible and was also shown for anti-adhesive functions of CDCP1 by Spassov et al. [32], the role of the extracellular domain seems to be complex. Truncation of the amino-terminal 216 amino acids leaves the first Cub domain intact, yet reduced focus formation. A truncation of 260 amino acids (not shown), mimicking the processed form identified by Brown et al. [11], and a truncation including the first Cub domain (ΔNT370) did not yield any foci. Especially the latter finding is surprising since most tumor derived cell lines contain a protein corresponding to ΔNT370 [24]. However, this result is supported by the data derived from the processing deficient mutant that has a higher capability to generate foci, is tyrosine phosphorylated and associates with Src (not shown). Thus, proteolytic processing apparently limits the potential of CDCP1 to overcome contact inhibition, and may rather function as a switch-off mechanism in normal cell physiology. These findings are differing from the results of He et al. [33] and Casar et al. [34] that proposed an essential role of CDCP1 proteolytic processing for phosphorylation, Src association and cell survival in DU145 cells. However, there are numerous other cell lines that also contain uncleaved CDCP1 that is tyrosine phosphorylated and associates with Src and PKCδ [24]. In fact, tyrosine phosphorylation can be specifically induced by binding of the antibody Cub1 to the uncleaved CDCP1 [35]. Law et al. [36] found that E-cadherin bound preferentially to the cleaved form of CDCP1 and speculated that this may be causal for the reduced invasion of some tumor derived cell lines. A further role of E-cadherin for cleaved CDCP1 phosphorylation and protein association should be evaluated. Of note, NIH3T3 cells do not express E-cadherin [37].

We did show that CDCP1 dimerizes and that the transmembrane domain is responsible for dimerization. In adherent keratinocytes, dimerization was not sufficient for tyrosine phosphorylation of CDCP1 since addition of ligating and not activating antibodies did not but binding of “activating antibodies” did induce tyrosine phosphorylation of CDCP1 [38]. Similarly, addition of Cub1-antibodies to HCT116 cells also induced CDCP1 tyrosine phosphorylation [35]. This indicates the requirement for binding to a specific epitope that forces CDCP1 into a conformation inducing phosphorylation, as is found for antibodies directed against receptor tyrosine kinases. Further, Alvares et al. [38] also reported the presence of CDCP1 in higher order clusters, supporting our findings shown in Figure 4. Together, these data suggest that CDCP1 per se is present as a dimer, but conformational change is required for activation of Src or other kinases.

Right after the transmembrane domain, there are two cysteine residues followed by a stretch of six lysine residues. Both cysteines did not contribute to CDCP1 dimerization but were essential for focus formation. One way this effect could be mediated is by palmitoylation and association with lipid rafts [14], bringing CDCP1 to a membrane domain that is enriched in other signaling proteins including the Src kinase. However, we have not been able to prove different detergent solubility for the CDCP1 wild type or cysteine mutant (data not shown). Alternatively, palmitoylation can protect from ubiquitination and degradation, as was described for the membrane protein TEM8 [39]. Failure to get palmitoylated may explain our difficulties to generate a cell line that permanently expresses the cysteine mutant to a similar extent as the wild type protein. However, we have not been able to upregulate the protein by proteasome inhibition or detect ubiquitin at CDCP1 in Western blotting, as it was described for TEM8.

The different CDCP1 mutants were expressed at similar levels in 293 cells. As an important aspect for comparison of transformation efficiency we also looked for expression in infected NIH3T3 cells. With the exception of the Cys-mutant, the CDCP1 point mutants were found at similar levels in these cell pools. The amino- and carboxyterminal deletion mutants, however, were consistently found expressed at a level between the Cys- and the other point mutants. Thus, the reduced number of foci generated by ΔNT217 may at least in part be due to a reduced protein level in infected cells whereas the failure of the other mutants to generate foci likely would not be essentially improved by an enhanced expression.

Mutation of the Src kinase also affected transformation efficiency. A mutant form of Src that was unable to be myristoylated did not support focus formation, while Src mutations inactivating the binding capacity of the SH2 (R177A) or SH3 (W120A) domains slightly enhanced it. In contrast, binding of Src to CDCP1 in overexpressing 293 cells depended mostly on the Src-SH2 but also to some extent on the SH3 domain, as demonstrated in the co-immunoprecipitation of CDCP1 and the corresponding Src mutants (Fig. 5). Surprisingly, myristoylation-defective Src was fully able to phosphorylate CDCP1, even though it bound to CDCP1 poorly. Thus, the intact Src-SH2 domain itself is not sufficient for association, but in addition a plasma membrane localization of Src is required. The strong phosphorylation of CDCP1 by the G2A-mutant is in contrast to recent findings of Patwardhan and Resh [40], showing that a lack of myristoylation reduces Src kinase activity about 3-fold for the substrate p130-Cas. However, the interaction of Src with the soluble p130-Cas versus the membrane localized substrate CDCP1 may be completely different. On the other hand, the binding of Src to Y734 of CDCP1 via its SH2 domain is required for phosphorylation of Y734 (wild type Src versus Src-R177A and CDCP1-Y734 as the only tyrosine). When Src is intact but cannot directly bind to Y734 of CDCP1 (wild type Src and CDCP1-Y734F) other tyrosines are still phosphorylated. These somewhat conflicting findings suggest the requirement of additional, not yet identified proteins participating in Src activation and CDCP1 phosphorylation.

We have begun to employ the focus formation assay to identify other proteins relevant for Src/CDCP1 mediated transformation. Triple infection of cells always led to a reduced focus formation, even when a virus was used that did not encode a cDNA (pLXSN-vector; not shown). Therefore, we only performed triple infection experiments in which the additionally expressed protein was available in an activating as well as in a not activating form. This was the case for PKCδ, for which we did not detect an effect on focus formation. The reason could be that the contribution of PKCδ is not reflected in this experimental system. Accordingly, Liu et al. [30] and Deryugina et al. [41] found only little effect of PKCδ on growth in soft agar.

Taken together, we have characterized important features of the CDCP1/Src mediated cellular transformation. In future experiments we should be able to further dissect the signaling pathway of CDCP1 in cellular transformation on the level of Src activation as well as the subsequent signaling steps.

## Materials and Methods

### Cell Lines, Plasmids and Antibodies

BOSC23, 293 and GP+E-86 cells were grown in Dulbecco’s modified Eagle’s medium (4.5 g/l glucose) containing 10% fetal calf serum and 2 mM L-glutamine. NIH3T3 cells were grown in Dulbecco’s modified Eagle’s medium with the same supplements and 1 g/l glucose. Bosc23, GP +E-86, NIH3T3 and 293 cells are available from ATCC.

For transient expression the vectors pRK5 (BD Pharmingen, Germany) or pcDNA3 (Invitrogen, Germany) containing an immediate early CMV promoter were used. To generate retroviruses, cDNAs were cloned into the vector pLXSN (BD Clontech, Germany). Point mutations or small deletions in the CDCP1 cDNA were generated using the Quikchange kit from Stratagene. Larger deletions were generated by overlap extension PCR.

An anti-Src rabbit polyclonal serum was directed against the carboxy-terminal 15 amino acids. Phosphotyrosine was detected with the antibody 4G10 (Millipore, Germany). Cub1 antibody for immunoprecipitation of CDCP1 has been described [42]. For immunoblotting, mab2666 from R&D Systems (Germany) detected the extracellular domain, whereas a rabbit antiserum was directed against the carboxy-terminal 15 amino acids (kind gift of H. Kalbacher). Secondary antibodies were horseradish peroxidase-coupled anti-rabbit or anti-mouse IgGs (Sigma, Germany).

### Lysis of Cells and Blotting Analysis

Transfections were performed using the method of Chen and Okayama [43]. Cells were lysed in lysis buffer (50 mM HEPES, pH 7.2, 150 mM NaC1, 1.5 mM MgC12, 1 mM EGTA, 10% glycerol, 1% Triton X-100, 2 mM phenylmethylsulfonyl fluoride, 10 µg/ml aprotinin, 100 mM NaF, 10 mM sodium pyrophosphate, and 1 mM sodium orthovanadate) and the lysates were cleared by centrifugation at 13 000 g for 5 min at 4°C. To the lysates, Laemmli buffer was added directly, or proteins were first immunoprecipitated. The immunoprecipitates were washed with HNTG buffer (50 mM HEPES, pH 7.2, 150 mM NaCl, 100 mM NaF, 1 mM sodium orthovanadate, 10% glycerol, 0.1% Triton X-100), and Laemmli buffer was added before the samples were boiled. The proteins were size-separated by SDS-polyacrylamide gel electrophoresis, transferred to nitrocellulose filters and analyzed by immunoblotting. Proteins were visualized with chemiluminescence (ECL, ThermoFisher, Germany).

### Focus Formation Assay

Virus containing supernatants from transfected Bosc23 cells were used to infect GP+E-86 cells in the presence of 6 µg/ml of polybrene. Individual clones were tested for virus secretion, good producers selected and cells expanded for production. For a focus formation assay, 50,000 NIH3T3 cells were seeded into a six-well dish and 18 h later infected in the presence of 6 µg/ml polybrene. On the next day, medium was changed to contain 4% fetal calf serum. Forty-eight h after infection, cells were trypsinized and seeded into a 10 cm dish in Dulbecco’s modified Eagle’s medium (1 g/l glucose) containing 4% fetal calf serum. The medium was changed three times a week for 3 weeks and then the cells were stained with crystal violet (0.5% crystal violet in 20% methanol). All focus formation assays shown are representative for at least 3 independent experiments.

## Supporting Information

Figure S1 **Expression profiles of wild type and mutant CDCP1 proteins.** NIH3T3 cells were infected with 300.000 viruses of each type, selected for G418 resistance, cell pools lysed and equal amounts of protein separated on SDS-PAGE. After protein transfer to nitrocellulose, the membranes were incubated with antibodies detecting the carboxy-terminus or the extracellular domain. Right panel: the upper arrow indicates the position for wild type CDCP1, the lower the position of mutant Δ702–836. Since mab2666 is human specific, proteins detected in the control lane represent unspecific cross-reactivities of the antibody. Ctrl - parental NIH3T3 cells
(TIF)

Figure S2 **Role of PKCδ for CDCP1 mediated transformation of NIH3T3 cells.** NIH3T3 cells were infected with the indicated viruses, grown and stained as described in Figure 1. (A) PKCδ, its inactive or constitutively active version do not affect Src/CDCP1 mediated transformation of NIH3T3 cells differently. The number of PKC isoform encoding viruses was similar. (B) In a different assay, generation of foci was compared between wild type and CDCP1-Stop-762 that does not bind PKCδ. Since the number of CDCP1-Stop-762 viruses used for infection was twice that of wild type CDCP1, the mutant is only half as effective in generating foci.
(TIF)

Figure S3 **Src mutants and transformation of NIH3T3 cells.** Cells were infected and stained as described. (A) Src-KA does not generate foci upon coinfection with CDCP1. Of note, the number of Src-KA encoding viruses was twice that of wild type Src. (B) Src mutants W120A and R177A have a higher potential to transform NIH3T3 cells.
(TIF)
