# Supplementary figures and images for: Structural Requirements for Cub Domain Containing Protein 1 (CDCP1) and Src Dependent Cell Transformation
Source: PLoS One. 2012 Dec 31;7(12):e53050. doi: 10.1371/journal.pone.0053050 (PMC3534080; doi:10.1371/journal.pone.0053050)

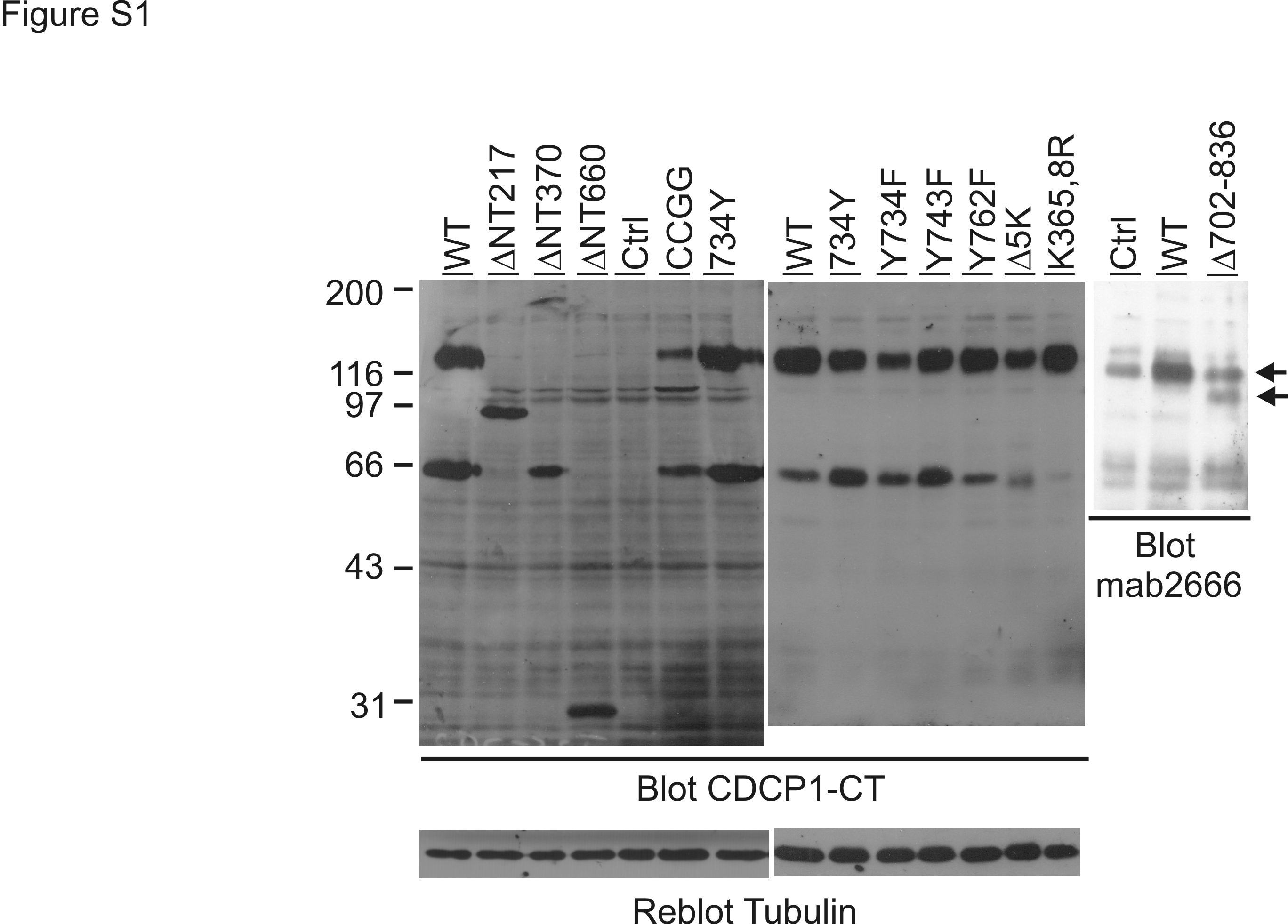

Supplement: Figure S1 — Expression profiles of wild type and mutant CDCP1 proteins. NIH3T3 cells were infected with 300.000 viruses of each type, selected for G418 resistance, cell pools lysed and equal amounts of protein separated on SDS-PAGE. After protein transfer to nitrocellulose, the membranes were incubated with antibodies detecting the carboxy-terminus or the extracellular domain. Right panel: the upper arrow indicates the position for wild type CDCP1, the lower the position of mutant Δ702–836. Since mab2666 is human specific, proteins detected in the control lane represent unspecific cross-reactivities of the antibody. Ctrl - parental NIH3T3 cells (TIF) [file pone.0053050.s001.tif]

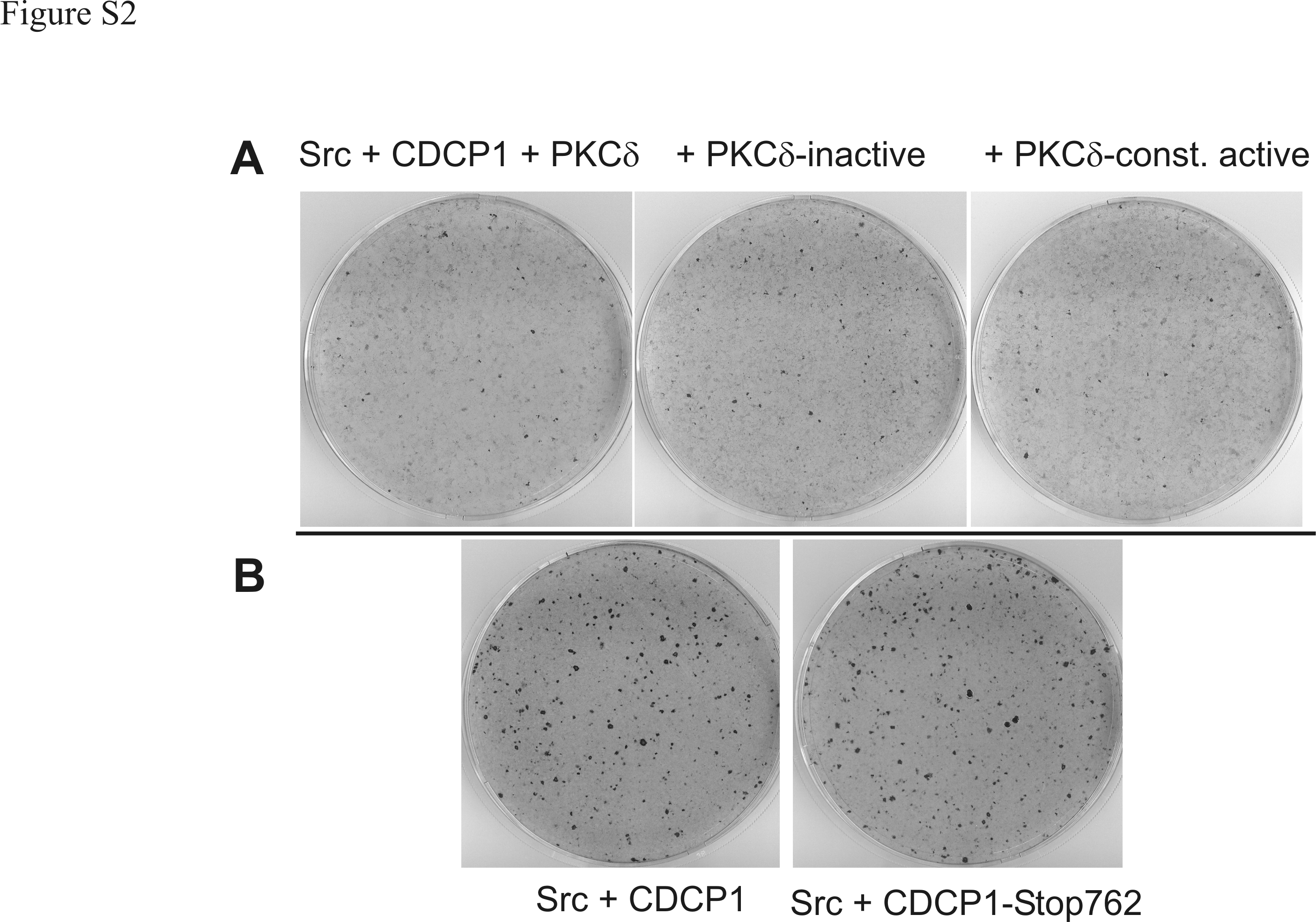

Supplement: Figure S2 — Role of PKCδ for CDCP1 mediated transformation of NIH3T3 cells. NIH3T3 cells were infected with the indicated viruses, grown and stained as described in Figure 1. (A) PKCδ, its inactive or constitutively active version do not affect Src/CDCP1 mediated transformation of NIH3T3 cells differently. The number of PKC isoform encoding viruses was similar. (B) In a different assay, generation of foci was compared between wild type and CDCP1-Stop-762 that does not bind PKCδ. Since the number of CDCP1-Stop-762 viruses used for infection was twice that of wild type CDCP1, the mutant is only half as effective in generating foci. (TIF) [file pone.0053050.s002.tif]

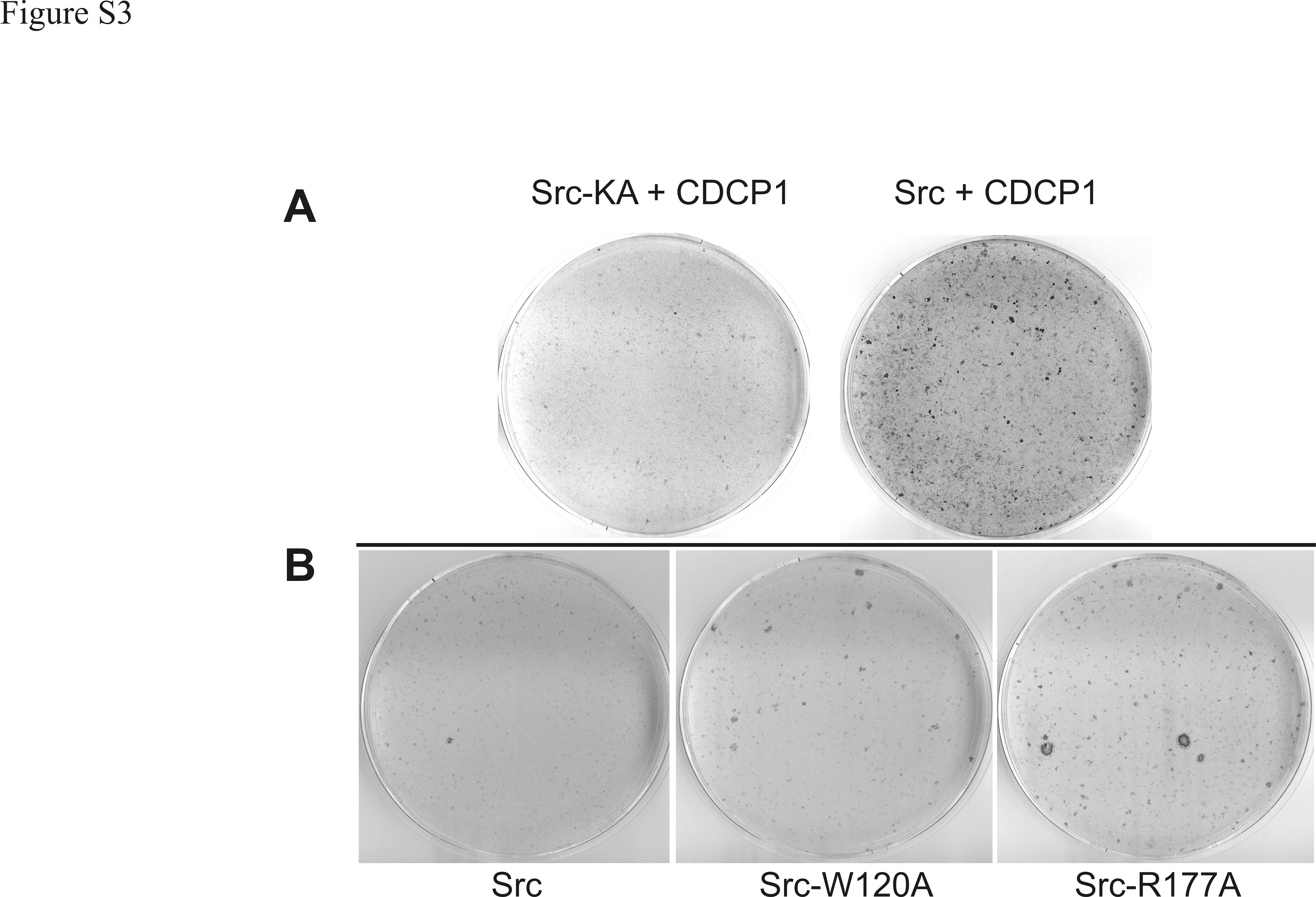

Supplement: Figure S3 — Src mutants and transformation of NIH3T3 cells. Cells were infected and stained as described. (A) Src-KA does not generate foci upon coinfection with CDCP1. Of note, the number of Src-KA encoding viruses was twice that of wild type Src. (B) Src mutants W120A and R177A have a higher potential to transform NIH3T3 cells. (TIF) [file pone.0053050.s003.tif]
